# The efficacy of cognitive behavioral therapy for cancer: A scientometric analysis

**DOI:** 10.3389/fpsyt.2022.1030630

**Published:** 2022-11-07

**Authors:** Chuanghao Lin, Huiting Tian, Lingzhi Chen, Qiuping Yang, Jinyao Wu, Zeqi Ji, Daitian Zheng, Zhiyang Li, Yanna Xie

**Affiliations:** ^1^Department of General Surgery, The Second Affiliated Hospital of Shantou University Medical College, Shantou, China; ^2^Department of Respiratory and Critical Care Medicine, The First Affiliated Hospital of Shantou University Medical College, Shantou, China

**Keywords:** cognitive behavioral therapy, cancer, Scientometrics, psychological, mechanisms

## Abstract

Cognitive behavioral therapy (CBT) is one of the most recognized psychological interventions to improve the overall quality of life of cancer survivors. To analyze current research trends in the field of the link between CBT and cancer and to provide potential future research directions, we conducted the scientometric analysis. The study was conducted on all documents in this field from 2012 to 2022 retrieved from Web of Science. Then Biblioshiny, VOSviewer software, and CiteSpace software were used for getting the information of article postings and citations, countries, institutions, journals, authors, and keywords. The number of documents about the link between CBT and cancer from 2012 to 19 July 2022, was 619, with 476 of articles and 143 of reviews. The number of annual publications has been fluctuating, with the highest number of publications in 2020. The country with the maximum number of publications and citations was the US. The University of Houston was the organization with the highest quantity of publications and total link strength (TLS). Psycho-Oncology was the most active journal in the field and has the highest h-index. Zvolensky MJ was the author with the highest quantity of publications. The most cited keywords were “Quality-of-life,” “Cognitive-behavioral therapy,” “Depression,” “Cognitive therapy” and “Breast-cancer.” And as evidenced by the keyword citations, the focus of this research area has gradually shifted to the mental health of patients and the underlying pathogenesis. The impact of CBT in cancer treatment is now well established and has gradually evolved toward symptom-specific treatment. However, the relationship between CBT and cancer has not been further developed. Future research is needed to be further developed in the identification of a generic formula for CBT in cancer and the exploration of mechanisms of CBT and cancer.

## Introduction

Cancer is one of the world’s most incurable diseases and has the second highest mortality rate after cardiovascular disease (1). According to statistics of GLOBOCAN 2020, 19.3 million new cancer cases and almost 10 million cancer deaths were indicated in 2020. By assuming the cancer incidence rate remains unchanged as in 2020, there will be 28.4 million new cancer cases worldwide in 2040, an increase of 47% from 2020 (2). With the development of early screening and innovative therapies such as personalized medicine and immunotherapy to prolong survival, the survival rate for cancer has increased significantly. However, this also brings with other aspects of problems. Long-term ongoing cancer treatment can take a huge physical toll and psychological burden on patients (1). Survivors may face months to years of cancer triggering and/or treatment-related symptoms such as fright of cancer recurrence, fatigue, insomnia, obesity, etc. which can adversely affect the quality of life of survivors (1, 3). Therefore, it is especially vital to monitor and ameliorate the adverse consequences of cancer treatment in the course of the chronic process of cancer therapy. Cognitive behavioral therapy (CBT) is a treatment approach that uses psychological and behavioral interventions to alter the patient’s dysfunction. Evidence suggests that CBT is the most effective psychological intervention to improve tiredness caused by cancer therapy and can make the quality of life of cancer survivors better (4–6).

CBT is a psychotherapeutic intervention that focuses on problem. It is an integration of behavioral interventions such as behavioral stimulation, exposure treatment, emotion regulation and relaxation training (7). It has evolved over more than 60 years from traditional face-to-face therapy to a diverse range of therapies such as internet-facilitated cognitive behavioral interventions. As an evidence-based treatment modality, CBT has been proven to be used in the treatment of a variety of psychiatric disorders such as depression, anxiety disorders, personality disorders, schizophrenia and more (8). Recently, the combination of CBT and medication is becoming more common in clinical practice and achieve greater efficacy, which will be one of the future directions of research and treatment.

There is a growing body of clinical researches on CBT and cancer, and most of them have confirmed the relationship between CBT and cancer. Although CBT is effective in monitoring and improving prognosis for cancer survivors, most survivors reported not discussing psychological interventions for cancer with their treatment providers, much less using them (8).

Therefore, we conducted a scientometric analysis of the relevant publications in the field and identified the current research. Our analysis aimed to get specific information on the publication profile of the field, such as publications and citations, top active countries, most productive authors, influential journals, hot topics and keyword analysis, identifying current issues that need to be addressed and predicting key directions for research in the future.

## Materials and methods

### Data collection

We searched all documents about the link between CBT and cancer on the Web of Science Core Collection (WoS) online databases. All entry terms related to “CBT” and “cancer” were searched as search themes through the medical subject headings (Mesh) of the PubMed website. The search input included the following: #1, ((((ALL = (”Cognitive Behavior Therapy”)) OR ALL = (”Cognitive Therapy”)) OR ALL = (”Cognitive Psychotherapy”)) OR ALL = (”Cognition Therapy”)) OR ALL = (”Cognitive Behavior Therapy”); #2, (((((((ALL = (cancer)) OR ALL = (carcinoma)) OR ALL = (neoplasm)) OR ALL = (adenocarcinoma)) OR ALL = (melanoma)) OR ALL = (adenocarcinoma)) OR ALL = (sarcoma)) OR ALL = (osteosarcoma); #3, #1 and #2. Then, time span of these publications was filtered from 2012 to 2022. The research was conducted on July 19, 2022. We searched for a total of 692 documents. After refining the types of documents to articles and reviews and restricting the language to English, 619 documents were retrieved, including 476 articles and 143 reviews. In total, 73 publications were excluded during the screening process: 9 articles not in English, 3 reviews not in English, 42 meeting abstracts, 9 editorial materials, 5 letters, 3 corrections, 1 biographical-items, and 1 book review.

### Data analysis

We applied the online analysis of WoS, bibliometrix, VOSviewer software, and CiteSpace software to perform a scientometric analysis of all retrieved articles. The online “Results Analysis” function of the WoS was initially used to access important information on the article topics, publication dates and types, research fields, countries, authors, institutions, journals, and free access for these publications. The WoS “Citation Report” function also allowed us to obtain additional information about the publications, like the total quantity of citations to articles, the specific quantity of citations per article, average quantity of citations per article and the trend in citations over time, etc.

Biblioshiny is the online analytics site of bibliometrix with powerful features for data analysis (version 4.2.1), one of which could be applied to perform a comprehensive scientometric analysis and to generate visual image of results (9). Therefore, we imported the raw files of the retrieved documents into this website and obtained related information about these publications, including time span, annual growth rate, number of sources, documents, and references, types of documents, authors, collaboration of authors, etc. In addition, other information analyzed on the Bibliometrix website would also be included in our scientometric analysis, including growth trend of annual publication outputs, and citations analysis, countries and organizations analysis (production), journal and research category analysis (journal sources, impact and dynamics), author analysis (authors’ contribution and impact), and keywords analysis. The three field diagrams showed the collaboration between different countries, authors and institutions, where more occupied areas proved a higher production of articles. Finally, the co-occurrence network of thematic evolution formed by keyword plus was an indicator to identify research hotspots.

VOSviewer (version 1.6.18), is a network analysis software, which can be used to construct network of authors or journals based on co-citation data and keyword network based on co-occurrence data. It can display bibliometric views of the results of different analyses of a set of data in four different views, such as label view, density view, cluster density view, scatter view (10). In our study, it was mainly used to demonstrate the strength of the links between different countries, different institutions and different authors and co-citation network of references and keywords.

CiteSpace V (version 6.1 R2) is a knowledge domain visualization tool, which can find the key points of the development of a research domain. It facilitates the identification of citation concentrations of literature and keywords in our analysis (11).

## Results

### Analysis of publication and citation

**FIGURE 1:**
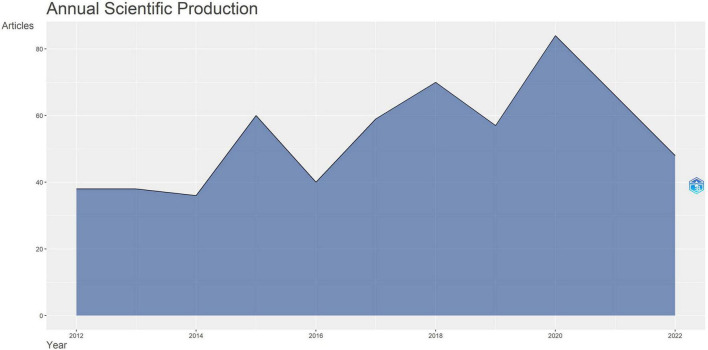
Annual article output of paper citations between 2012 and 2022 in this research area.

The all number of publications about the link between CBT and cancer that we retrieved from WoS from 2012 to 2022 was 619. 2020 was the year with the largest number of publication output (84, 13.57%). Production of annual publications was on an increasing trend from 38 publications (6.19%) in 2012 to 84 publications (13.57%) in 2020, but the overall trend seemed to be unstable between 2012 and 2022 (Figure 1). The period 2012–2014 was a relatively stable period with a steady annual publications of around 38 documents. The quantity of publications has fluctuated since 2014. Over the 7 years from 2014 to 2022, annual production has repeatedly risen and fallen a total of three times, which indicated that the overall development of the field is not mature enough. The annual production showed an increasing trend during 2014–2015, 2016–2018, and 2019–2020, while it shows a decreasing trend during 2015–2016, 2018–2019, and 2020–2022. The annual growth rate of annual scientific output is 2.36%, the highest in 2015 (66.67%) and the lowest in 2018 (18.64%). In 2020, annual production reached a maximum and exceeded 80 documents.

**FIGURE 2:**
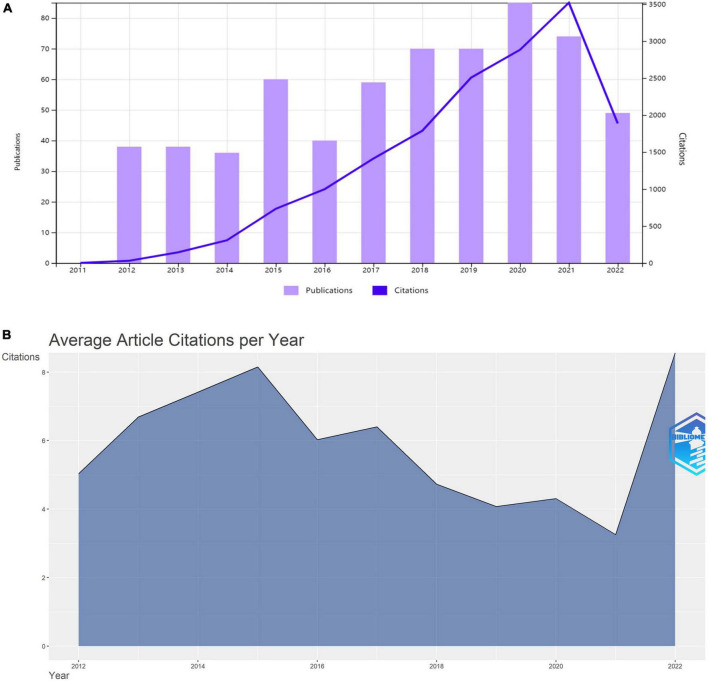
Trends in the overall quantity and average number of citations to articles per year from 2012 to 2022. **(A)** Trends in the overall quantity. **(B)** Average number of article citations.

An article’s scientific impact can be reflected by the number of citations (12). According to the citation report of WoS, there were 12,506 citing articles among all the retrieved publications, including 335 self-citations and 12,171 without self-citation (97.32% of all citations). The sum of all article citations between 2012 and 2022 was 16,213, including 15,254 no self-referencing, which accounted for 94.08% of all citations. Meanwhile, annual article citations of articles showed an upward trend, from 31 in 2012 to 3,519 in 2021 (Figure 2A). The average quantity of citations per article was 26.19. Figure 2B demonstrates annual trend of average article citations from 2012 to 2022. 2015 was the year with the maximum average number of article citations at 8.2. There was an upward trend before 2015 and a downward trend after 2015.

Supplementary Table 1 shows details of the top 10 most quoted papers within the study on CBT and cancer, which was integrated through the specific information from all the documents presented on the Biblioshiny website. Among them, the highly cited articles could indicate the current research focus in the field. These top 10 articles were mainly published between 2012 and 2017, with the most cited article being published in 2013. Of these ten articles, 30% were published in 2013 and 30% in 2014, and one in each of the remaining years. The most cited article was a clinical review on psychology (13), with a total of 998 citations (13). The article “How do mindfulness based cognitive therapy and mindfulness based stress reduction improve mental health and wellbeing? A systematic review and meta-analysis of mediation studies” published by Gu et al. (14) was cited 744 times (14). The work “cancer related fate mechanisms, risk factors, and treatments” written by Bower (15) was the third-ranked article (cited 579 times) (15). In total, 3 studies were cited more than 500 times, representing 0.50% of all papers; 6 papers (1.01%) were cited more than 300 times and 28 papers (4.70%) were cited more than 100 times. However, no article has been cited more than 1,000 times since its publication.

**FIGURE 3:**
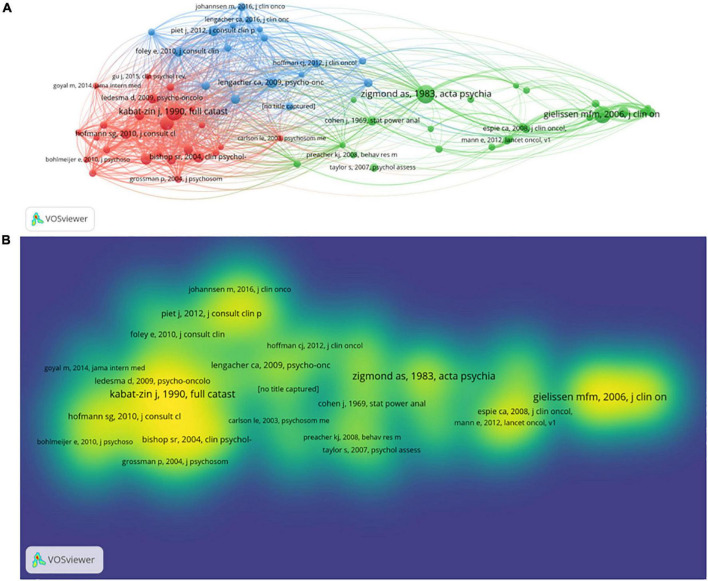
Co-citation network diagram of the cited references with a maximum of citations of 20 times for these documents. **(A)** Co-citation network diagram between references. **(B)** Co-citation density diagram for references.

Figure 3 is the co-citation network diagram of cited literature of these publications crafted by VOSviewer. After selecting 20 as the lowest number of citations for the cited literature, 66 references met the requirement. The size of the node was related to the quantity of reference citations with a larger node meaning more references cited (Figure 3A). In Figure 3A, we could observe that there were three clusters of the cited references, red, blue and green, with 25 items in the red cluster, 24 in green and 17 in blue. A cluster with a larger number of items means it is more attractive in the research area. The citations of reference could also be seen from Figure 3B. The yellower color indicated more co-citations. In Figure 3, the article of Zigmond and Snaith (16) had the highest citations as reference, with 68 citations (16). The other four of the top five most cited as reference were Gielissen et al. (17) (64 citations), Irving and Segal (18) (50 citations), and Bishop et al. (19) (41 citations), Lengacher et al. (20) (40 citations). Moreover, we counted the top 25 references with the highest intensity of citations by CiteSpace software (Supplementary Figure 1). Among these 25 papers, 20% (5/25) developed citation bursts in 2012, with the second-ranking in 2018 (4/25, 16%) and then third in 2015, 2016, 2017, and 2020, respectively (3/25, 12%). The paper written by Cillessen (21) was the reference with the strongest burst (strength = 4.95), which had the citation bursts from 2020 to now. Additionally, 6 references (24%) were cited consecutively until 2022, while 5 references (20%) were separately cited until 2017 and 2020.

### Analysis of countries and institutions

**FIGURE 4:**
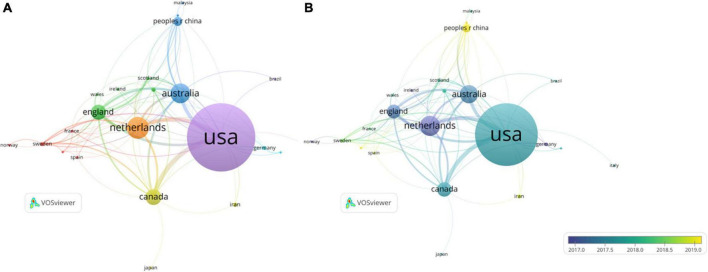
Co-authorship analysis of countries in the area. **(A)** Co-authorship network diagram between countries with over five documents. **(B)** Co-authorship overlap diagram between countries with over five documents.

The analysis of countries and organizations is a comparative analysis of the quantity of papers and article citations for each country and institution, which can show the current distribution of articles in this field. The 619 documents we retrieved were mainly from 44 countries or regions. Table 1 shows the top 10 countries with the maximum articles in the area. The US was the most active country among these 44 countries or regions, with a total of 276 publications (44.59% of all). Next up were the Netherlands (89 publications, 14.38%), Australia (82 publications, 12.92%), Canada (65 publications, 10.50%) and England (62 publications, 10.02%). Likewise, The United States leaded the way in terms of citations of publications, with 8,194 citations. It was followed by Canada (2,661 citations), Netherlands (2,596 citations) and England (2,226 citations). We also could compare the quantity of publications in different countries by the size of the points in Figure 4. We then analyzed the strength of co-authorship connections with over 5 publications between different countries (Figure 4). The thickness of the line connecting the circles is related to the total connection strength (TLS) between countries. The top 5 countries with the highest TLS were the US (TLS = 107), Australia (76), England (71), Netherlands (68), and Canada (68) (Table 1 and Figure 4). From 2018 to 2019, some developing countries began to publish articles in this field and made great achievements, especially China. China published the first paper in the field in 2018 and has now published a sum of 37 articles in 2022, making it the sixth most published country. At the same time, China was increasing the intensity of its partnerships with other countries (TLS = 22).

**TABLE 1:** Top 10 countries with the maximum articles in the area.

Country	Total publications	Total citations	Total link strength
USA	276	8,194	107
Netherlands	89	8,194	68
Australia	80	2,127	76
Canada	65	2,661	68
England	62	2,226	71
China	37	416	22
Denmark	18	956	23
Iran	18	118	4
Sweden	17	201	31
Germany	16	562	14

In general, a total of 929 organizations have been implicated in the 619 documents. The University of Houston has produced the most number of publications (66 publications, 10.66%), then was followed by the University of Texas MD Anderson Cancer Center (65 publications, 10.50%), Radboud University Nijmegen (53 publications, 8.56%), the University of Sydney (25 publications, 4.04%), the University of Amsterdam (24 publications, 3.88%) (Table 2).

**TABLE 2:** Top 10 most productive organizations in the research area.

Organization	Total publications	Total citations	TLS
University of Houston	66	596	108
University of Texas MD Anderson cancer center	65	572	105
Radboud University Nijmegen	53	1,322	84
University of Sydney	25	614	39
University of Amsterdam	24	450	68
Kings College of London	18	216	36
Memorial Sloan Kettering Cancer Center	18	540	26
Vrije Universiteit Amsterdam	17	262	61
University of Groningen	16	387	37
University of Queensland	13	257	51

**FIGURE 5:**
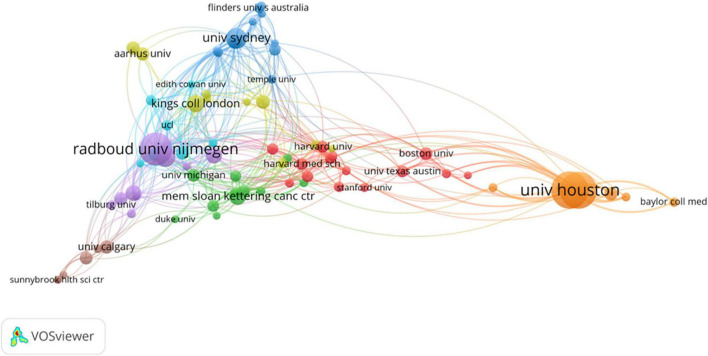
Co-authorship network diagram between institutions in the area.

We launched an analysis of co-authorship relationships for organizations with more than five publications, yielding results for a total of 76 organizations. Then, we obtained a plot of the relationship between the TLS between different institutions (Figure 5). Sorted by TLS, the top 5 organizations were the University of Houston (108), the University of Texas MD Anderson Cancer Center (105), Radboud University Nijmegen (84), the University of Amsterdam (68), and the Vrije Universiteit Amsterdam (61).

### Analysis of journal and research category

**FIGURE 6:**
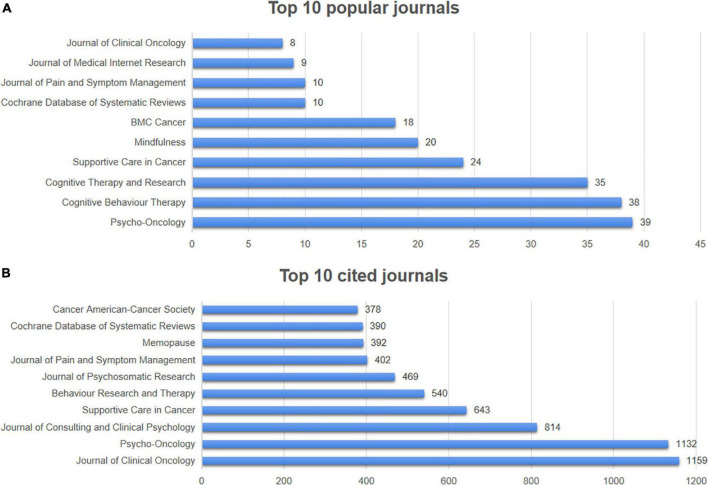
Top 10 popular journals and cited journals. **(A)** Top 10 popular journals regarding the number of related articles. **(B)** Top 10 popular journals regarding the number of citations.

The 619 papers were published in 264 different sources of journals. Figure 6 shows the top 10 popular journals regarding the number of related articles and citations. There was a total of 211 articles published by the top 10 popular journals, which accounting for 34.09% of all. *Psycho-Oncology* was the most active journals of papers, with 39 publications. *Cognitive Behavior Therapy* ranked second (38 publications), and was followed by *Cognitive Therapy and Research* (35 publications), *Supportive Care in Cancer* (24 publications) and *Mindfulness* (20 publications). Four journals of the top 5 popular journals mainly dedicated to psychology (Figure 6A). According to Figure 6B, *Journal of Clinical Oncology* contributed the maximum quantity of publication citations with 1,159. *Psycho-Oncology* was the second most cited journal, which had 1,132 citations. And it was followed by *Journal of Consulting and Clinical Psychology* (814 citations), *Supportive Care in Cancer* (643 citations) and *Behavior Research and Therapy* (540 citations).

The h-index, the largest number of articles published by a researcher or journal which have been cited h or more times, would be used to measure the academic achievements of a researcher or journal based on both quantity and impact (22). Sequenced by h-index, the top 5 journals were *Psycho-Oncology* (h-index = 17), *Supportive Care in Cancer* (h-index = 12), *BMC Cancer* (h-index = 11), *Cognitive Therapy and Research* (h-index = 11), *Mindfulness* (h-index = 11) (Table 3). *Psycho-Oncology*, *BMC Cancer*, *Cognitive Therapy and Research, Supportive Care in Cancer* and *Mindfulness* have remained active in the field during the years, especially *Psycho-Oncology*, which has been number one in terms of cumulative publications since 2012. *Cognitive Behavior Therapy* was constantly enhancing its influence in this research area, which attempted to publish articles in this field after 2014 and had become the second largest journal of cumulative publications by 2022 (Supplementary Figure 2).

**TABLE 3:** Top 10 highest impact journals by source in the field.

Source	h-index	g-index	m-index	PY start
Psycho-oncology	17	30	–	–
Supportive care in cancer	12	18	1.091	2012
BMC cancer	11	17	1.000	2012
Cognitive therapy and research	11	12	1.100	2013
Mindfulness	11	15	1.222	2014
Cochrane database of systematic reviews	9	10	0.818	2012
Cognitive behavior therapy	8	13	–	–
Journal of clinical oncology	8	8	0.727	2012
Journal of pain and symptom management	8	9	0.727	2012
Journal of medical internet research	6	9	1.000	2017

**FIGURE 7:**
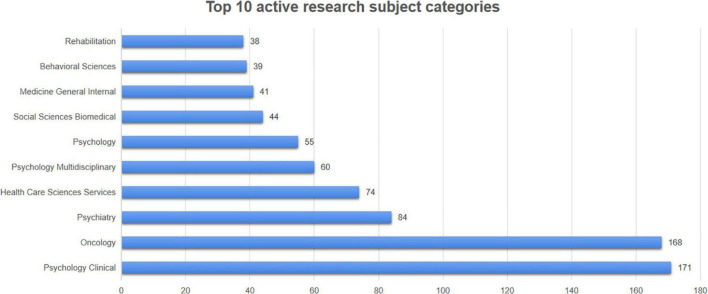
Top 10 active research subject categories.

These 619 papers cover a total of 61 research categories. Figure 7 demonstrates the top 10 active research subject categories. “Psychology Clinical” (171 publications), “Oncology” (168 publications), “Psychiatry” (84 publications), “Health Care Sciences Services” (74 publications) and “Psychology Multidisciplinary” (60 publications) were the top 5 research subject categories.

### Analysis of author

According to the main information of 619 documents, a total of 2,864 authors were involved, including 20 authors for single-author articles and 2,844 authors for multi-author articles. Zvolensky MJ was the most active author in this field, with 62 publications (10.02% of all), which far exceeded that of the latter authors. The authors immediately following were Bleijenberg G (*n* = 21, 3.39%), Garey L (*n* = 20, 3.23%), Knoop H (*n* = 20, 3.23%) and Hunter MS (*n* = 17, 2.75%) (Table 4). Only one author has published more than 50 articles. Four authors have published more than 20 articles, and 17 authors have published over 10 articles. We created a figure of the top ten most-published authors in the field for each year (as shown in Supplementary Figure 3), with the size of the dots representing the articles published in that year and the intensity of the color of the dots representing the total number of citations per year. Zvolensky MJ’s publications were low until 2014 but began to increase rapidly after 2014 and remained more active in the latter 5 years. Bleijenberg G has published more in the first few years and less in the last 5 years, while Garey L has published less in the first few years and more in the last 4 years. The number of articles published by Knoop H and Hunter MS has maintained a steady trend in recent years.

**TABLE 4:** Top 10 productive authors in the area of cognitive behavioral therapy and cancer.

Authors	Articles	h-index	g-index	m-index	PY-start
Zvolensky MJ	62	11	19	–	2012
Bleijenberg G	21	12	20	1.091	2012
Garey L	20	6	13	0.857	2016
Knoop H	20	12	20	1.091	2012
Hunter MS	17	9	15	0.818	2012
Gielissen MFM	14	9	14	0.818	2012
Prins JB	14	8	14	0.727	2012
Van der lee ML	13	6	6	0.667	2014
Goedendorp MM	12	9	12	0.818	2012
Savard J	12	5	11	0.455	2012

Top 10 productive authors in the area of cognitive behavioral therapy and cancer.

As with the analysis of journal, the h-index was applied to analyze the scholarly achievement and value of each author’s paper. Only three of the top ten authors had an h-index of more than 10. Both Bleijenberg G and Knoop H had the highest h-index of 12 and was followed by Zvolensky MJ (h-index = 11), Hunter MS (h-index = 9), Gielissen MFM (h-index = 9), and Goedendorp MM (h-index = 9). The m-index was used to eliminate the effect of age and experience between authors, taking into account the differences in the start of publication and the quantity of papers published by different authors (23). Gallagher MW was the author with the highest m-index (m-index = 1,250). Bleijenberg G and Knoop H ranked No. 2, with the m-index of 1,091. The rest of the authors had an m-index either equal to 1,000 or less than 1,000.

The top 8 authors with the most times cited of their publications in the research scope of CBT and cancer were listed in Table 5. The articles written by Zachariae R had the most citations of 557. Zvolensky MJ ranked second, with 509 citations. And then there were Thewes B (343 citations), Carlson LE (302 citations) and Beatty L (291 citations). In addition, we analyzed the TLS between authors who published at least five articles. The top five authors were Zvolensky MJ (TLS = 121), Bleijenberg G (TLS = 120), Garey L (TLS = 89), Goedendorp MM (TLS = 77), and Knoop H (TLS = 76).

**TABLE 5:** Top 8 authors with the most times cited of their publications in the research scope of cognitive behavioral therapy and cancer.

Author	Documents	Citations	Total link strength
Zachariae R	9	557	63
Zvolensky MJ	62	509	121
Thewes B	7	343	29
Carlson LE	7	302	33
Beatty L	9	291	34
Bleijenberg G	17	255	120
Garland SN	7	243	13
Sharpe L	6	240	16

**FIGURE 8:**
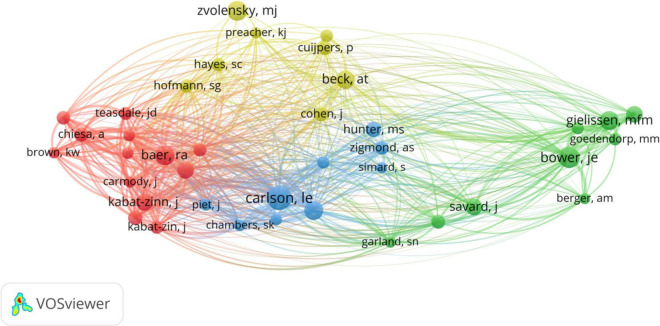
Co-citations network diagram between authors with more than 50 citations.

Co-citation analysis of authors was performed in Figure 8 by VOSviewer software. A total of 39 authors were selected with more than 50 citations. In co-citation analysis, a total of six authors had a TLS of over 1,000. The top five authors with the maximum connection strength were Carlson LE (TLS = 1,744), Baer RA (TLS = 1,504), Lengacher CA (TLS = 1,234), Segal ZV (TLS = 1,106) and Kabat-zinn J (TLS = 1,023).

**FIGURE 9:**
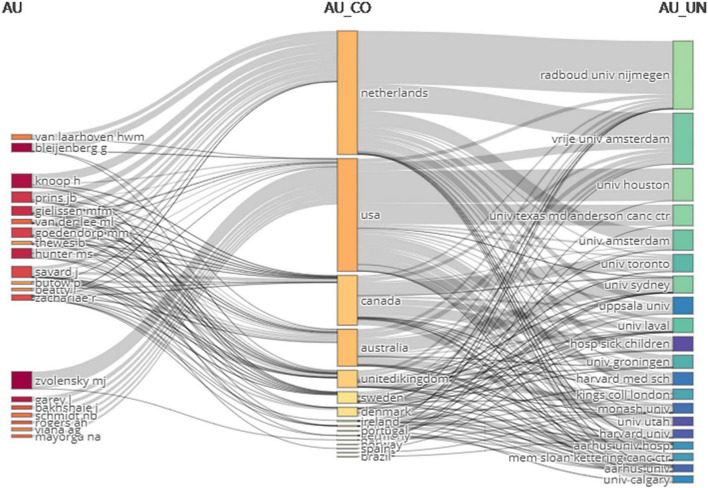
The three-field diagram of the collaboration between different countries **(middle)**, authors **(left)** and organizations **(right)** (Occupied area represents the number of articles issued and the density of line indicates the closeness of cooperation).

Figure 9 is a three-field diagram, which indicates the collaboration among different countries (middle), authors (left), and organizations (right). The occupied area of the region in the figure represents the number of documents issued by a country, institution or author, and the density of line indicates the closeness of cooperation between them. The United States was the country with the strongest collaboration between organizations and authors in the field. The University of Houston and the University of Texas MD Anderson Cancer Center were responsible for nearly half of the articles published in the field in the United States. And the author with the strongest collaboration with the U.S. was Zvolensky MJ. The Radboud University Nijmegen and the Vrije Universiteit Amsterdam were two of the organizations with the most cooperations with other countries. As for the authors, most of them preferred to work with one country, and only some had cooperations with more than one country or institution. The total number of cooperation between Zvolensky MJ and other countries was the largest, and Knoop H was the second.

### Analysis of keywords

**FIGURE 10:**
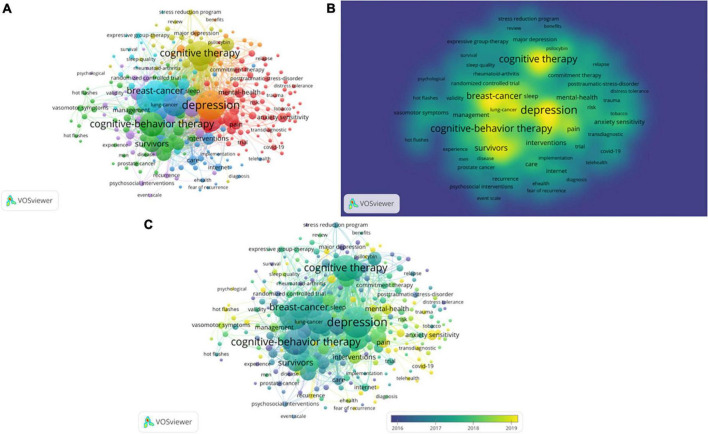
Co-occurrence analysis of keywords. **(A)** Keywords network map of related documents (7 clusters). **(B)** Keywords density map based on frequency of occurrence. **(C)** Keywords overlap map based on average year of publication.

**FIGURE 11:**
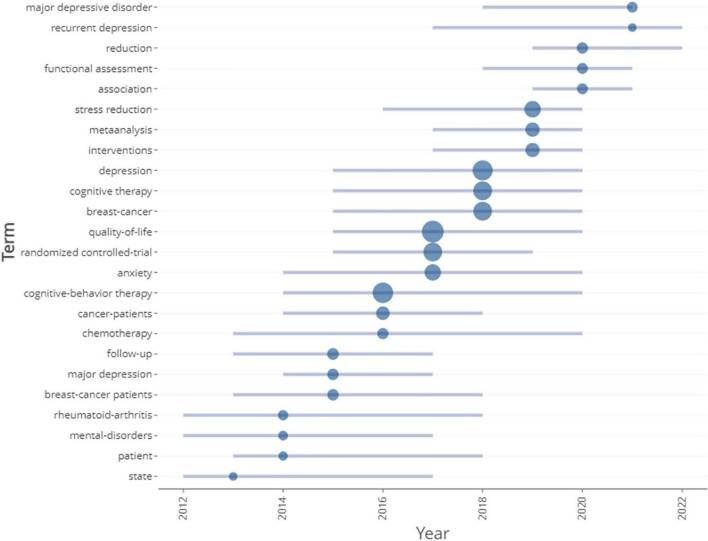
Trends in keyword development over the years.

After selecting five times as the minimum number of occurrences of the keyword, we obtained 273 keywords. The clustering analysis function of VOSviewer software would be used to divide 273 items into several clusters. There was a total of seven different colored clusters in the network visualization map (Figure 10). The relevance of keywords in clusters of the same color was higher. The size of the node in the Figure 10A and the degree of yellow in the Figure 10B represented the frequency of keyword occurrences. The top 10 frequent words in the research scope of CBT and cancer were listed in Table 6 and their rate of growth and number of cumulative occurrences per year were showed in Supplementary Figure 4. There was a total of six keywords with the occurrence of more than 100 times. “Quality-of-life” was the most frequently used keyword with 193 occurrences and was followed by “Cognitive-behavioral therapy” (156 occurrences), “Depression” (149 occurrences), “Cognitive therapy” (127 occurrences), “Breast-cancer” (123 occurrences), and “Randomized controlled-trial” (119 occurrences). In term of TLS, the top 5 keywords were “Depression” (TLS = 1,971), “Quality-of-life” (TLS = 1,689), “Cancer” (TLS = 1,506), “Cognitive therapy” (TLS = 1,387) and “Cognitive-behavioral therapy” (TLS = 1,239). Figure 10C is the overlay visualization map which shows the average time of keyword appearance. The change in color reflects the time of occurrence of the keyword. Figure 11 indicates the trends of keyword development over the years. The length of the line indicates the duration of the keyword, the year in which the dot appears means that the keyword appears most often at that time, and the larger the dot, the more often it appears. Through Figures 10C, 11, we could find out a gradual shift in the focus of the research field from the adverse effects and prognosis of cancer therapy and the role of CBT to the mental health of patients and the mechanisms of morbidity.

**TABLE 6:** Top 10 frequent words in the research scope of cognitive behavioral therapy and cancer.

Words	Occurrences	Total link strength
Quality-of-life	193	1,689
Cognitive-behavior therapy	156	1,239
Depression	149	1,971
Cognitive therapy	127	1,387
Breast-cancer	123	1,133
Randomized controlled-trial	119	985
Anxiety	78	1,201
Stress reduction	78	705
Survivors	78	759
Validation	73	674

**FIGURE 12:**
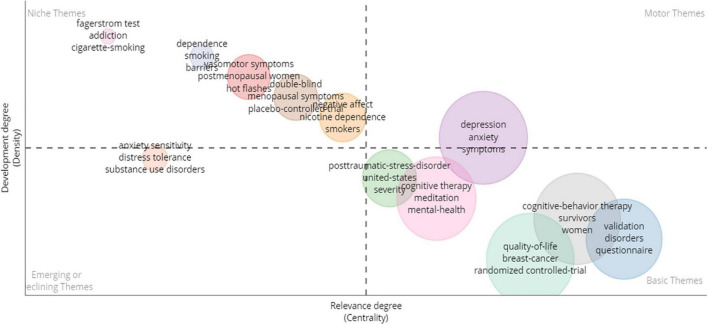
Keywords plus of four quadrants. Motor themes (first quadrant); Niche themes (second quadrant); Emerging or declining themes (third quadrant); Basic themes (fourth quadrant).

Using the data provided by the Bibliometrix website, we analyzed the keywords plus that appear in the literature cited in the article. Figure 12 shows a four-quadrant diagram with the different keyword degree of relevance and development. The bottom horizontal axis represents the degree of relevance (centrality), with centrality representing relevance of the keyword to the field of study. The leftmost vertical axis represents the degree of development (density), which represents the degree to which the keyword has been studied or developed in the field. The four quadrants are: (First quadrant) Motor themes: the themes in this region have the best development trend and are most relevant to the area. (Second quadrant) Niche themes: the development of themes in this region is good, but it is lack of connection with other articles in this field. (Third quadrant) Emerging or declining themes: these themes are less well developed and related in this field of study. It is possible that these themes have just appeared or are in the process of disappearing. (Fourth quadrant) Basic themes: the themes are strongly related to this field, but are hardly developmental. They are may be some basic concepts or knowledge. From Figure 12, the keywords were divided into numerous clusters with different color. We could find that the purple cluster’s keywords (“Depression,” “Anxiety,” “Symptoms”) were in the first quadrant, which meant they were closely related to the field and were particularly important, while the yellow-brown cluster’s keywords (“Anxiety sensitivity,” “Distress tolerance,” “Substance use disorders”) were in the third quadrant and were not indicative for the moment. Compared to the small number of clusters present in quadrant one and there, there were many clusters present in quadrant two and four. The clusters in the second quadrant, such as orange clusters (“Negative effect,” “Nicotine dependence,” “Smokers”) and red clusters (“Vasomotor symptoms,” “Postmenopausal women,” “Hot flashes”), were highly developmental, but had little connection with articles in this area. The clusters in the fourth quadrant, like gray cluster (“Cognitive-behavioral therapy,” “Survivors,” “Women”), and pink cluster (“Cognitive therapy,” “Meditation,” “Mental-health”), had a strong link to the researches but were poorly developed. In addition, both the green and orange clusters were close to the center, indicating a high degree of keyword plus relevance in both clusters.

**FIGURE 13:**
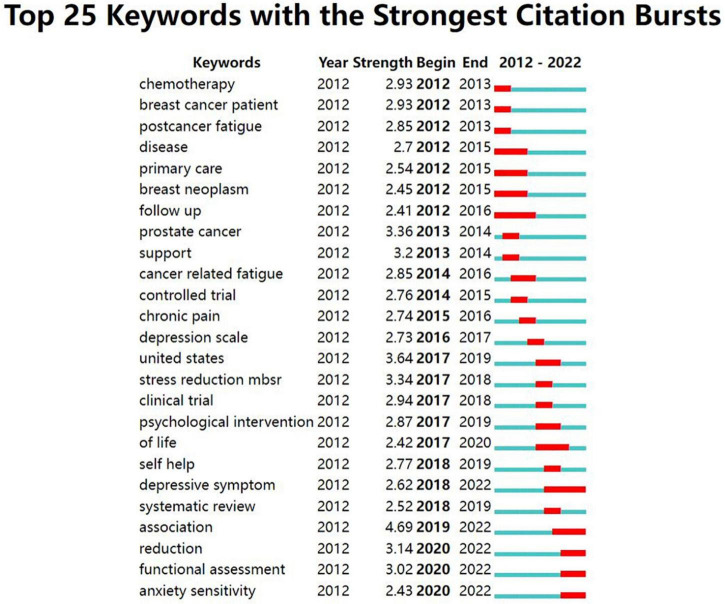
Top 25 keywords with highest citations bursts in this area.

We conducted a citation burst analysis of the keywords by CiteSpaceV software. Keyword citation burst analysis allows for a more detailed study of keyword citation duration and citation burst intensity. Figure 13 displays the top 25 keywords with the strongest citation burst. The outbreak of keyword citations appearing in different years represents trends in the research field for that year. The citations burst of these keywords started from 2012 to 2020. Among the top 25 keywords, 28% (7/25) began to appear citation explosion in 2012, the second was 2017 (20%, 5/25), and the third was 2018 and 2020 (15%, respectively, 3/25). “Association” had the highest citation burst intensity at 4.69 and the citation burst lasted from 2019 to 2022. Other keywords with citation burst strength of over 3 were “United States” (Citation burst strength = 3.64), “Prostate cancer” (3.36), “Stress reduction: Mindfulness-based stress reduction (MBSR)” (3.34), “Support” (3.2), “Reduction” (3.14), “Functional assessment” (3.02). There were 5 keywords for which the citation burst lasts until 2022. The last 12 keywords have been consistently cited in recent years, proving that they were the focus of recent research in the field, particularly “Depressive symptom,” “Association,” “Reduction,” “Functional assessment” and “Anxiety sensitivity,” which will continue to be active beyond 2022.

## Discussion

This is a scientometric analysis of articles on the link between CBT and cancer published in 2012–2022 by using Bibliometrix, VOSviewer software, and CiteSpace software. The number of articles published in this field from 2012 to July 19, 2022 was 619. The number of papers showed an overall increasing trend until 2020 with the highest in 2020. In total, there were 16,213 citations across all the articles searched. The average number of citations per article was 26.19, with the average number of citations showing an upward trend until 2015 and a downward trend after 2015. This may be related to the fact that the relationship between cancer and CBT has not been further developed. There is now a larger body of literature demonstrating the role of CBT in cancer treatment. With the development of early screening and innovative therapies, the mortality rate of cancer has been greatly reduced and the number of cancer survivors has increased significantly. But cancer survivors may face months to years of cancer triggers and/or treatment-related symptoms such as fright of cancer recurrence, fatigue, insomnia, obesity, etc. after long-term cancer treatment. The application of CBT can be very effective in alleviating these problems (23–26). Cognitive behavioral therapies have evolved to target a specific symptom, such as CBT for insomnia (CBT-I) (27), CBT for fatigue (CBT-F) (28), and so on. However, this is only a small step forward in improving the precision of CBT. The relationship between CBT and cancer has still not been groundbreaking on this basis.

In total, 44 countries or regions contributed to the 619 articles in the country and institution analysis. The US was the largest contributor to the field with 276 articles, accounting for nearly 45% of the total. The top two institutions in terms of number of publications, the University of Houston and the University of Texas MD Anderson Cancer Center, are both located in the United States, which could demonstrate the importance and contribution of the US to the field. The US was a pioneer in the field of CBT and cancer, but other countries were also making breakthroughs in the field. A prime example of this was China. It took only 4 years for China to go from publishing its first article to being the sixth most published country. The trend of Chinese publication themes showed the progress of Chinese research in this field, from the early days of studying the relationship between CBT and cancer, to exploring the refinement of CBT for specific adverse effects caused by specific cancer treatments, and focusing more on patients’ psychological health and living conditions (29, 30). In addition, the biggest breakthrough of China in this area was the inclusion of the most representative Chinese elements such as acupuncture and Tai Chi in the research process (31–33), and the study of the effectiveness of these approaches in cognitive behavioral disorders. This is a completely new direction and deserves further research.

Among the top five journals with the maximum number of publications, four were psychological. Psycho-Oncology was the most common theme of research in the field. From the beginning, the psychological theme and the cancer theme were not overlapping, but have now evolved into psycho-oncology, which combines both themes and ensures a high volume of publications in the field.

Zvolensky MJ was the most active author in the field, with about three times the number of articles published by the second-placed Bleijenberg G. He was also the third highest author in the h-index apart from Bleijenberg G and Knoop H. His articles focused on the role of the relationship between emotional states and psychopathology on certain human behaviors, such as smoking and suicide, and were mainly aimed at younger people (34–36). Although Zvolensky MJ’s research has not focused on the link between CBT and cancer, his research on the link between psychopathology and behavioral and emotional regulation was very thorough and revealed the potential mechanisms behind the development of mental health problems such as anxiety, distress and fatigue, such as distress tolerance (DT) and anxiety sensitivity (AS) (34, 37), which could provide a basis for the role of CBT in the treatment of poor mental health caused by long-term cancer treatment. And this has greatly guided the direction of subsequent research in the field.

There were two relatively novel points of research in this field during the literature search. The first was the use of “Acute cancer CBT,” which was different from conventional CBT, and the other is that there was numerous research on the role of CBT in breast cancer, but very little on the role of CBT in other cancers.

The emergence of acute CBT is mainly due to the fact that conventional CBT is primarily indicated for the treatment of cancers with a chronic course and most cognitive behavioral studies are driven by established protocols where the patient is stable and has a stable course of treatment. In contrast, CBT for acute cancers can be very different. The instability and uncertainty of acute cancers tend to cause greater psychological shock to patients than chronic cancers. This impact can be understood as a breakdown of psychological defenses due to difficulties in accepting, over-interpreting, misunderstanding, etc. of cancer. The difficulty and challenge of CBT for acute cancer lies in its unpredictable acute cancer setting. CBT is primarily characterized by crisis response to acute cancer settings. However, the life-threatening urgency as well as the unpredictability and adverse effects of cancer treatment can add to the crisis atmosphere, thus making the implementation of CBT more difficult. Levin and Applebaum (38) developed a cognitive behavioral treatment framework applicable to the integration of acute healthcare settings and applied it to acute cancer environment. In summary, empathy, coping models and psychopharmacology are core elements of CBT for acute cancer (38, 39). In addition, perceptions of death, and mortality, are important components of acute cancer CBT. Although the model of CBT for acute cancer is different from that for chronic cancer, it has been researched and developed over many years for clinical application and is recommended as an essential training skill for clinicians (39).

The link between breast cancer and CBT is also one of the main directions in the field at present. Breast cancer was the most prevalent cancer among women and the second leading cause of cancer death in women (40, 41). Evidence suggested that depression was one of the most common psychiatric disorders in breast cancer patients, with over half (58%) of patients within the female breast cancer population experiencing mild depressive symptoms and 38% experiencing major depression (42, 43). Mindfulness-based cognitive therapy (MBCT) was currently an effective intervention for psychiatric disorders associated with cancer treatment. Luberto et al. (44) described the theoretical rationale for MBCT for cancer recurrence and provided case examples (44). MBCT could guide the emotional changes of breast cancer patients, reduce their psychological disorders, and then reduce the risk of anxiety and depression. In addition, in the article “Predictors and moderators of outcomes in MBCT intervention for early breast cancer patients” (45), the authors aimed to discover the predictors and moderators of outcomes in MBCT for early breast cancer patients and explore the optimal course of treatment that produces significant outcomes (45). It can be argued that the role of MBCT in the treatment of breast cancer is now becoming clearer and represents a vital milestone in the palliative care of breast cancer patients (46). Similarly, CBT has also played a significant role in the treatment of other cancers, such as ovarian and prostate cancers, colorectal cancer and others (47–49). However, research in this area is currently limited to these specific cancers, and the role of CBT in the treatment of other cancers is not yet known. Therefore, whether CBT has the same efficacy in other cancers should be one of the directions for future research. A general formula for CBT in cancer treatment will be established in the future.

From the results of the keyword analysis, current research has focused on the role of CBT in adverse effects such as fatigue, pain, anxiety and depression following long-term cancer treatment. And these studies have mainly focused on a few specific types of cancer, such as breast cancer. It was clear that the research of CBT in cancer is precise but not extensive, and it fails to involve more kinds of cancer. However, this deficiency should be remedied and a general formula for CBT in cancer will be available in the future. Almost every research study is inseparable from the exploration of mechanisms. This is also true for the field of CBT and cancer. However, there was currently little research in this area that involves cellular or molecular mechanisms, which is a relatively new direction of research. In addition, the frequent occurrence of keywords such as “Mental-health,” “Stress-disorder” and “Anxiety sensitivity” in recent years indicated that the current research has gradually shifted from pure disease treatment to treatment targeting patients’ physical and mental health. Therefore, the exploration of the mechanisms between CBT and cancer, as well as the psychological state of cancer patients, will also be the focus of research in the future.

The article “The modulatory role of internet-supported MBCT on extracellular vesicles and psychological distress in people who have had cancer: a protocol for a two-armed randomized controlled study” written by Pereira et al. (50) was an important milestone in the development of molecular mechanisms in this field (50). It revealed the possible molecular mechanisms by which psychological interventions can improve cancer prognosis. The extracellular vesicles and their contents promote cancer growth, clarity and metastasis through intercellular communication and signal transmission and can therefore be used for cancer screening and diagnosis. The authors evaluated the concentration of biochemical markers in the blood of the body, such as interleukin, tumor necrosis factor, C-reactive protein, etc., telomerase activity, which is a marker related to cancer recovery, and cancer-related antigens, and then detected the impact of MBCT intervention on the overall immune response, finally measured the changes of extracellular vesicles in the central nervous system (50). This article started with the substances that promote the growth and metastasis of cancer, analyzed whether psychological intervention measures can affect the changes of these substances, and then revealed some potential molecular mechanisms of psychological intervention in cancer treatment. Although this is an initial attempt to investigate the molecular mechanisms in the field of CBT and cancer, it is a huge step forward in the development of the field. Future research into the cellular molecular mechanisms of CBT in cancer treatment is set to flourish.

## Conclusion

The relationship between CBT and cancer has become increasingly clear over time, and its role in ameliorating adverse effects following long-term cancer treatment has become indispensable. This scientometric analysis was completed to analyze 619 documents on the scope of CBT and cancer over the period 2012–2022 through Bibliomerix, VOSviewer, and CiteSpace software. The study focuses on a detailed analysis of countries, institutions, authors, keywords, etc., providing a large number of visual figures and tables and revealing the relationships between them. These analyses also provide insights into the current focus of research in the field and possible future research directions. Most of the research in this area has focused on exploring the role of CBT in the treatment of fatigue, depression, anxiety, fear of cancer recurrence and other adverse reactions following long-term cancer treatment, and on refining CBT for specific symptoms. The role of CBT for more cancers, general formulations of CBT in cancer, and the exploration of mechanisms between CBT and cancer will be the focus of future research in this area.

## Data availability statement

The raw data supporting the conclusions of this article will be made available by the authors, without undue reservation.

## Author contributions

CL, HT, and LC involved in the overall design of the research, critically examined, and improved the article. CL was the writing, data collection, and data analysis for this manuscript. HT and LC provided instruction on the use of relevant analytical applications, literature collection, and data interpretation. QY, JW, ZJ, DZ, ZL, and YX contributed to the discussion of the article results. All authors checked and agreed on the final submitted version and contributed to the completion of this article.

## Supplementary material

Supplementary Figure 1 Top 25 references with highest citations bursts in this area.

Supplementary Figure 2 Source growth over the years related to this research scope.

Supplementary Figure 3 Authors’ production of this field over time.

Supplementary Figure 4 Cumulative occurrences of top 10 frequent words in the field of cognitive behavioral therapy and cancer.

## Abbreviations

CBT: Cognitive behavioral therapy
WoS: Web of Science
TLS: Total link strength
CBT-I: Cognitive behavioral therapy for insomnia
CBT-F: Cognitive behavioral therapy for fatigue
MBCT: Mindfulness-based cognitive therapy.
