# Supplementary material for: The efficacy of cognitive behavioral therapy for cancer: A scientometric analysis
Source: Front Psychiatry. 2022 Nov 7;13:1030630. doi: 10.3389/fpsyt.2022.1030630 (PMC9676684; doi:10.3389/fpsyt.2022.1030630)
Supplement: Supplementary file 1 [file Table_1.docx]

**Supplementary Table 1** Top 10-cited documents in the research scope of cognitive behavioral therapy and cancer with corresponding authors, publication year, journal, volume, issue, page, total citations.

| Authors | Article | Journal | Year | Vol | Issue | Page | TC |
| --- | --- | --- | --- | --- | --- | --- | --- |
| Khoury B; Lecomte T; Fortin G; Masse M; Therien P; Bouchard V; Chapleau MA; Paquin K; Hofmann SG | Mindfulness-based therapy: A comprehensive meta-analysis | Clinical Psychology Review | 2013 | 33 | 6 | 763-771 | 998 |
| Gu J; Strauss C; Bond R; Cavanagh K | How do Mindfulness-Based Cognitive Therapy and Mindfulness-Based Stress Reduction Improve Mental Health and Wellbeing? A Systematic Review and Meta-Analysis of Mediation Studies | Clinical Psychology Review | 2015 | 37 | -- | 1-12 | 744 |
| Bower JE | Cancer-related fatigue—mechanisms, risk factors, and treatments | Nature Reviews Clinical Oncology | 2014 | 11 | 10 | 597-609 | 579 |
| Spijkerman MPJ; Pots WTM; Bohlmeijer ET | Effectiveness of online mindfulness-based interventions in improving mental health: A review and meta-analysis of randomised controlled trials | Clinical Psychology Review | 2016 | 45 | -- | 102-114 | 380 |
| Andersen BL; Derubeis RJ; Berman BS; Gruman J; Champion VL; Massie MJ; Holland JC; Partridge AH; Bak K; Somerfield MR; Rowland JH | Screening, Assessment, and Care of Anxiety and Depressive Symptoms in Adults With Cancer: An American Society of Clinical Oncology Guideline Adaptation | Journal of Clinical Oncology | 2014 | 32 | 15 | 1605-1619 | 379 |
| Stuenkel CA; Davis SR; Gompel A; Lumsden MA; Murad MH; Pinkerton JV; Santen RJ | Treatment of Symptoms of the Menopause: An Endocrine Society Clinical Practice Guideline | Journal of Clinical Endocrinology & Metabolism | 2015 | 100 | 11 | -- | 329 |
| Gotink RA; Chu P; Busschbach JJV; Benson H; Fricchione GL; Hunink MGM | Standardised Mindfulness-Based Interventions in Healthcare: An Overview of Systematic Reviews and Meta-Analyses of RCTs | Plos One | 2015 | 10 | 4 | -- | 289 |
| O'reilly GA; Cook L; Spruijt-Metz D; Black DS | Mindfulness-based interventions for obesity-related eating behaviours: a literature review | Obesity Reviews | 2014 | 15 | 6 | 453-461 | 255 |
| Piet J; Würtzen H; Zachariae R | The Effect of Mindfulness-Based Therapy on Symptoms of Anxiety and Depression in Adult Cancer Patients and Survivors: A Systematic Review and Meta-Analysis | Journal of Consulting and Clinical Psychology | 2012 | 80 | 6 | 1007-1020 | 246 |
| Parsons CE; Crane C; Parsons LJ; Fjorback LO; Kuyken W | Home practice in Mindfulness-Based Cognitive Therapy and Mindfulness-Based Stress Reduction: a systematic review and meta-analysis of participants’ mindfulness practice and its association with outcomes | Behaviour Research and Therapy | 2017 | 95 | -- | -- | 224 |
