# Supplementary figures and images for: The efficacy of cognitive behavioral therapy for cancer: A scientometric analysis
Source: Front Psychiatry. 2022 Nov 7;13:1030630. doi: 10.3389/fpsyt.2022.1030630 (PMC9676684; doi:10.3389/fpsyt.2022.1030630)

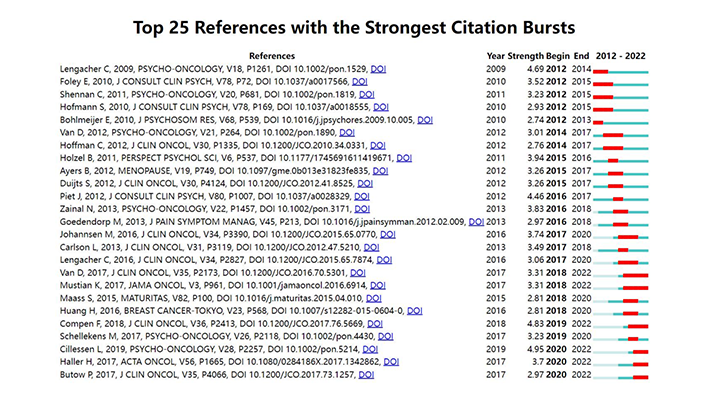

Supplement: Supplementary Figure 1 — Top 25 references with highest citations bursts in this area. [file Image_1.TIF]

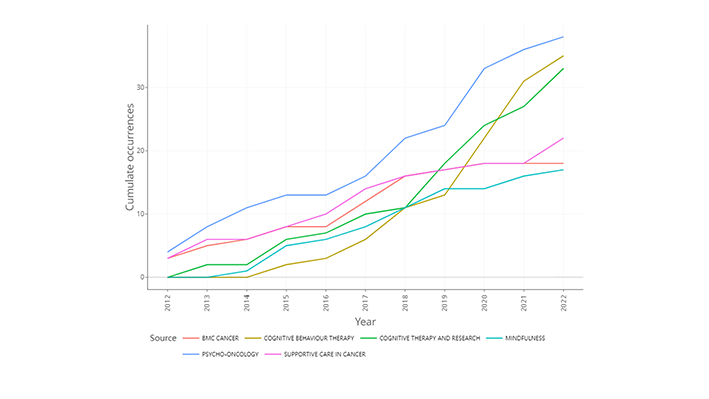

Supplement: Supplementary Figure 2 — Source growth over the years related to this research scope. [file Image_2.TIF]

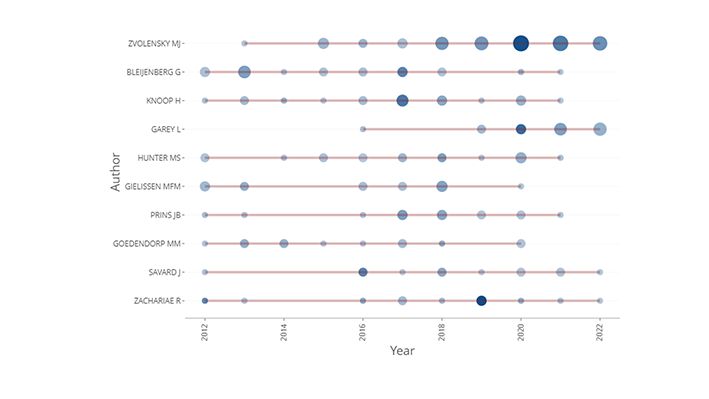

Supplement: Supplementary Figure 3 — Authors’ production of this field over time. [file Image_3.TIF]

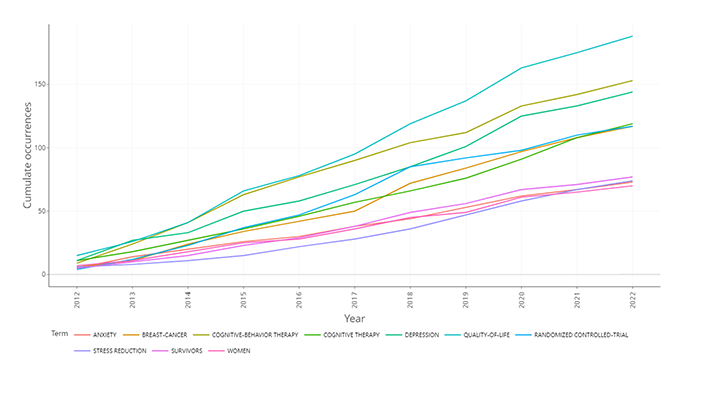

Supplement: Supplementary Figure 4 — Cumulative occurrences of top 10 frequent words in the field of cognitive behavioral therapy and cancer. [file Image_4.TIF]
